# C-756-03. Development of a Comprehensive Pediatric Outcome Measure: The School-Aged Life Impact Burn Recovery Evaluation Instrument

**DOI:** 10.1093/jbcr/irag033.047

**Published:** 2026-04-06

**Authors:** Madeleine McGwin, Pengsheng Ni, Sophia McLaughlin, Agnes McDonough, Ludwik K Branski, Tina L Palmieri, Mary D Slavin, J Michael Murphy, Frederick J Stoddard Jr, Jeffrey C Schneider, Lewis E Kazis, Colleen M Ryan

**Affiliations:** Shriners Children's - Boston, Boston, MA; Boston University School of Public Health, Boston, MA; Shriners Children's - Boston, Boston, MA; Shriners Children's - Boston, Boston, MA; University of Texas Medical Branch and Shriners Hospital for Children, Galveston, Texas and Shriners Hospital for Children, Galveston, TX; University of California Davis Health, Shriners Children’s - Northern California, Sacramento, CA; Massachusetts General Hospital Brigham - Spaulding, Boston, MA; Massachusetts General Hospital, Boston, MA; Harvard Medical School at Massachusetts General Hospital, Boston, MA; Spaulding Rehabilitation Hospital, Mass General Brigham, Harvard Medical School, Boston, MA; Boston University School of Public Health, Boston, MA; Massachusetts General Hospital, Harvard Medical School, Boston, MA

## Abstract

**Introduction:**

Assessing health-related quality of life in school-aged children is a continued challenge to the burn field. A new instrument, designed to monitor key aspects of a child’s life following burn injury, is needed to address this challenge. This instrument can support recovery and rehabilitation in children living with burns during this critical stage of growth. The objective of this study is to examine the psychometric properties of the School-Aged Life Impact Burn Recovery Evaluation (SA-LIBRE) Profile Computer Adaptive Test (CAT), an innovative instrument developed to assess burn survivors 5 to 12 years of age based on parent responses.

**Methods:**

Responses to the field-tested SA-LIBRE Profile (195 items) were recorded using frequency and ability response scales. Scores were coded from 0 to 4, with higher scores indicating better functioning. Factor analysis identified the unidimensional domains, or scales. Item Response Theory (IRT)-based analyses established item parameters and calibrated item banks for each scale. CAT simulations were conducted to estimate mean scores for each scale. The simulated CAT score and full item bank scores were compared based upon the score range, ceiling and floor effects, and marginal reliability.

**Results:**

The sample included 416 parents of burn survivors. The mean age of the child was 8.5 ± 2.4 years (SD), and 54.3 % were male. The mean total body surface area burned was 8.7 %, with an average time since burn of 4.5 years. An eight-factor solution emerged: Functional Impact of Burn Symptoms (17 items), Upper Extremity (15 items), Lower Extremity (11 items), Psychosocial (22 items), Resilience (6 items), Body Image (13 items), Peer Support (10 items), and School (6 items). Upper and Lower Extremity functioning scales were analyzed separately for children with burns to the hands or arms (n = 288) and burns to the thigh, calf or feet (n = 171), respectively. CATs were developed for each scale, except for Resilience, Peer Support, and School, due to the small number of items in these item banks. Correlations between the CATs and full-item banks ranged from 0.94 to 0.99 (*p*<.05). Marginal reliabilities for the scales were credible after removing subjects performing at the floor and ceiling of the respective metrics and ranged from 0.61 to 0.87.

**Conclusions:**

The School-Aged LIBRE, a psychometrically sound assessment for children 5 to 12 years of age, includes 8 scales that assess key aspects of recovery for children after a burn injury.

**Applicability of Research to Practice:**

The eight scales of the School-Aged LIBRE will be made available for use in research and clinical practice.

**Funding for the study:**

This work was supported by Foundation Funding (Grant# 79138, 79 136, and 79 145) and in part by the National Institute on Disability, Independent Living, and Rehabilitation Research (Grant# 90DPBU0008).

